# Uncovering multi-level mental healthcare barriers for migrants: a qualitative analysis across China, Germany, Netherlands, Romania, and South Africa

**DOI:** 10.1186/s12889-024-19046-z

**Published:** 2024-06-14

**Authors:** Alina Ioana Forray, Ovidiu Oltean, Saskia Hanft-Robert, Rowan Madzamba, Andrian Liem, Barbara Schouten, Christine Anthonissen, Leslie Swartz, Răzvan Mircea Cherecheș, Sanna Higgen, Brian J. Hall, Mike Mösko

**Affiliations:** 1 Faculty of Political, Administrative and Communication Sciences, Center for Health Innovation, Babeș-Bolyai University, Cluj-Napoca, Romania; 2 Department of Community Medicine, Discipline of Public Health and Management, Iuliu Hațieganu University of Medicine and Pharmacy, Cluj-Napoca, Romania; 3 Department of Medical Psychology, University Medical Center Hamburg Eppendorf, Hamburg, Germany; 4 Department of Psychology, Stellenbosch University, Stellenbosch, South Africa; 5 Jeffrey Cheah School of Medicine and Health Sciences, Monash University, Selangor, Malaysia; 6 Department of Communication Science, Amsterdam School of Communication Research (ASCoR), University of Amsterdam, Center for Urban Mental Health, Amsterdam, The Netherlands; 7 Department of General Linguistics, Stellenbosch University, Stellenbosch, South Africa; 8 Postgraduate Program for Bio-Behavioral Integrative Medicine (UBBMed), Babeș-Bolyai University, Cluj-Napoca, Romania; 9 Center for Global Health Equity, NYU Shanghai, Shanghai, China; 10 Department of Applied Human Sciences, University of Applied Sciences Magdeburg-Stendal, Stendal, Germany

**Keywords:** Mental Health, Migration, Health Policy, Communication barriers, Healthcare System barriers, Qualitative research, Healthcare disparities

## Abstract

**Background:**

Forced displacement is a significant issue globally, and it affected 112 million people in 2022. Many of these people have found refuge in low- and middle-income countries. Migrants and refugees face complex and specialized health challenges, particularly in the area of mental health. This study aims to provide an in-depth qualitative assessment of the multi-level barriers that migrants face in accessing mental health services in Germany, Macao (Special Administrative Region of China), the Netherlands, Romania, and South Africa. The ultimate objective is to inform tailored health policy and management practices for this vulnerable population.

**Methods:**

Adhering to a qualitative research paradigm, the study centers on stakeholders’ perspectives spanning microsystems, mesosystems, and macrosystems of healthcare. Utilizing a purposive sampling methodology, key informants from the aforementioned geographical locations were engaged in semi-structured interviews. Data underwent thematic content analysis guided by a deductive-inductive approach.

**Results:**

The study unveiled three pivotal thematic barriers: language and communication obstacles, cultural impediments, and systemic constraints. The unavailability of professional interpreters universally exacerbated language barriers across all countries. Cultural barriers, stigmatization, and discrimination, specifically within the mental health sector, were found to limit access to healthcare further. Systemic barriers encompassed bureaucratic intricacies and a conspicuous lack of resources, including a failure to recognize the urgency of mental healthcare needs for migrants.

**Conclusions:**

This research elucidates the multifaceted, systemic challenges hindering equitable mental healthcare provision for migrants. It posits that sweeping policy reforms are imperative, advocating for the implementation of strategies, such as increasing the availability of language services, enhancing healthcare providers’ capacity, and legal framework and policy change to be more inclusive. The findings substantially contribute to scholarly discourse by providing an interdisciplinary and international lens on the barriers to mental healthcare access for displaced populations.

## Background

By the end of 2022, global forced displacement had reached a staggering 112 million individuals due to reasons spanning from persecution and conflict to severe human rights infringements and disruptions in public order. Breaking down the figures, refugees accounted for 35.3 million. Furthermore, 62.5 million internally displaced persons, 5.4 million asylum-seekers, and another 5.2 million people necessitate international protection, emphasizing the need for migration-responsive global health policies. Out of the displaced populations, 76% were hosted by low- and middle-income nations, while higher-income countries only provided asylum to 20% of the total [1, 2]. WHO’s report on refugee and migrant health delineates the adverse health outcomes this population frequently experiences, driven by subpar living, access to healthcare, and working conditions [3].

Migration is a key determinant of health, profoundly influencing healthcare access and interactions with health systems. The inclusivist approach defines “migrant” as an umbrella term and consistent with the International Organization for Migration’s definition [4]. This term encompasses all forms of movement, whether internal or international, temporary or permanent, and for a variety of reasons. It includes legally defined categories, as well as those not specifically defined under international law.

In contrast, the residualist approach distinguishes between migrants and refugees, acknowledging that refugees face unique challenges and legal statuses [5]. Refugees, as a specific subgroup within the broader category of migrants, are more likely to have experienced trauma and exhibit higher rates of mental illness compared to other migrants [6, 7]. Attitudes towards refugees and migrants also differ, with macro-level factors significantly influencing perceptions of refugees [8]. The historical separation of the refugee and migrant categories has implications for their protection and access to durable solutions [9].

Despite these distinctions, both groups share certain health needs, including communicable and non-communicable diseases, mental health issues, and social problems [10, 11]. Migrants and refugees face common challenges such as language and cultural barriers, low health literacy, social exclusion, and unfamiliarity with host country health systems, leading to suboptimal primary care usage and increased reliance on emergency services [12, 13]. Mental healthcare provision for migrants and refugees is particularly challenging due to complex barriers and polarized attitudes among healthcare professionals [14]. Both groups are vulnerable to language and cultural barriers and social exclusion, exacerbating their pre- and post-migratory trauma, anxiety-related disorders, stress, and depression [15, 16]. Factors such as trauma, lack of social support, and inadequate housing further hinder their adaptation and acquisition of necessary social and cultural capital in host countries [17]. Structural barriers to healthcare include long waiting lists, financial constraints, lack of knowledge about the healthcare system, language barriers, and poor trust [18–20]. To enhance the mental well-being of migrants and refugees, it is crucial to offer tailored services appropriate at different stages of resettlement. Economic stability should be improved in alignment with social welfare benefits throughout the resettlement journey. As migrants and refugees become more settled, interventions should address feelings of isolation and stress factors related to adapting to a new life in the host country [21].

In this study, we adopted an inclusivist approach, aiming to examine access to mental healthcare services regardless of individuals’ legal status, motivations to migrate, migratory status, and migratory experiences while taking into consideration the distinctions and similarities in accessing mental healthcare for both groups in a variety of settings.

Migrant settlement and integration are complex and nonlinear processes where individuals acquire the necessary cultural, social, and educational capital to adapt and thrive in the host society [22]. While the concept of integration is debated and contested [23], most scholars agree that acquiring country-specific skills and social capital are essential for accessing economic opportunities, housing, healthcare, and education [24, 25]. This process is often hindered by challenges faced both during migration and throughout settlement in the destination country [26, 27].

Access to healthcare for migrants who do not speak the primary languages used in host countries’ healthcare systems is compromised in various ways. This includes misdiagnosis, higher rates of non-adherence to healthcare advice and treatments, cultural mismatches between patients’ understanding of illness and the healthcare system’s understanding, and legal and bureaucratic difficulties [10, 28, 29]. These issues come into sharper focus in the context of mental health care provision among migrants, where clear communication between a clinician and a patient is crucial for effective care [30] and where trauma-related mental health problems are prevalent but often overlooked [31]. In this context, questions of trust in the healthcare system, cultural appropriateness, stigmatization, and social exclusion are significant concerns that need to be addressed to improve the quality of care for migrants [32–34].

Language barriers affect access to healthcare services and the outcome, as many migrants are unable to communicate in the official language of the host country [35]. To overcome this, migrants are often in difficult situations, having to revert to relatives to provide informal interpretation during patient encounters [36, 37]. Practices of informal interpreting can breach the confidentiality of the medical act, hamper the trust in the healthcare system, and weaken the effectiveness of medical treatment [38]. Strategies like cultural mediation, professional interpretation, translation of health information, collaboration between different agencies, guidance, and training for healthcare providers have been implemented to address communication barriers for refugees and migrants in healthcare settings. Incident reporting systems can also be promoted to identify problems with strategy implementation and finding solutions [39].

One WHO report identifies several models of care deployed by countries to adapt healthcare systems for refugees and migrants. The review acknowledges that healthcare worker behavior is influenced by the overarching healthcare system and aims to contextualize the settings and models under which they interact with refugee and migrant populations [40]. WHO’s report on the health of refugees and migrants highlights two crucial gaps: the lack of comparable, cross-country data and the absence of migratory status-specific data within global health datasets [3]. In addition, The Global Competency Standards for Health Workers in Refugee and Migrant Health developed by WHO underscores the necessity of a skilled and adaptable health workforce to meet the diverse needs of displaced populations. These standards aim to ensure a minimum level of competency tailored to the unique health challenges faced by refugees and migrants, such as language barriers, cultural differences, and restricted access to mainstream healthcare [41].

The aim of our study is to conduct a comprehensive qualitative analysis to uncover the barriers migrants face in accessing mental healthcare services in five distinct settings: Germany, the Netherlands, Romania, China, and South Africa. This research focuses on understanding the impact of language barriers, alongside other cultural and institutional obstacles, within the healthcare systems of both well-resourced and more modestly resourced countries. Through semi-structured interviews with a diverse group of stakeholders, including healthcare providers, policymakers, administrative staff, and professionals from migrant support organizations, our objectives are twofold: (1) To identify and categorize the specific barriers to access and treatment of adult migrants within mental health services in both well-resourced healthcare systems and those with more modest resources; (2) To explore how language barriers exacerbate these challenges and affect healthcare access and treatment outcomes for both patients and healthcare providers. This nuanced understanding will contribute to the formulation of policies and practices that enhance the accessibility and quality of mental healthcare for migrants, ultimately improving public health outcomes for this vulnerable group.

This study examines the cross-cultural context of five different countries, namely Germany and the Netherlands (high-income countries and common migrant destinations), Romania (a high-income country in Europe emerging as a migrant destination), and two upper-middle-income countries, China and South Africa (both of which host substantial migrant populations). By focusing on countries across Europe, Asia, and Africa, we explore barriers to care beyond the commonly studied European settings [26, 42, 43]. Our study can broaden the narrative on migrant health needs and enrich our understanding of the challenges that migrants face in accessing healthcare, as well as knowledge of the diverse experiences of migrants and the complexities of their interactions with healthcare systems. Our approach highlights the crucial role played by diverse varieties of barriers, especially language discordance, that hinder access to mental health for all individuals who are navigating their lives in a new country, regardless of their motives for migration or the intricate details of their migration experience.

Firstly, in all the studied countries, language barriers stand as a significant issue affecting migrants’ healthcare. In the Netherlands, 10% of residents predominantly use non-Dutch languages at home, exacerbating healthcare challenges [44], while Germany, with Europe’s largest foreign-born population, faces similar difficulties; many migrants lack proficiency in German even after prolonged residency [45]. Similarly, Romania’s rapidly growing migrant population from Southeast Asia often struggles with language barriers [46]. South Africa’s linguistically diverse migrant populace [47] and Macao’s surge in migrant workers highlight a similar trend [48].

Secondly, current healthcare systems in these countries offer insufficient or fragmented solutions to address the barriers faced by migrants. For example, budget cuts in the Netherlands led to a decline in formal interpretation services [49, 50]. In Germany, the lack of multilingual professionals and insufficient interpreter coverage limits migrants’ access to mental healthcare [51]. Romania’s healthcare system often relies on informal interpreters, usually accredited social workers from NGOs, leading to inconsistency and potential inaccuracies in the translation [46]. South Africa and Macao (China) lack formal healthcare interpreting services altogether, causing service delivery complications [47, 48, 52].

Thirdly, there have been reported disparities in access to mental healthcare for migrants across these countries. Germany provides extensive mental health services through outpatient care by psychiatrists, neurologists, and psychotherapists and inpatient care in specialized hospitals, while the availability of ambulatory psychotherapeutic care has been recognized as a key problem in mental healthcare in Germany, with long waiting times for therapy and significant differences between regions [53]. For refugees and asylum seekers, Germany employs a phased care model where initial care at reception centers is limited to basic healthcare. After 6–12 weeks, access is restricted to acute and emergency care, with full healthcare access granted only to those with long-term residence permits (12–48 months) [40]. Similarly, in the Netherlands, a stepped-care model with general practitioners as gatekeepers is in place, with recent legislation enhancing support for residence permit recipients but excluding those in the process of obtaining a permit [54, 55]. The mental healthcare system in Romania is predominantly hospital-centric, with limited outpatient services [56]. Outpatient psychotherapeutic services are rarely covered through statutory insurance and often present bureaucratic challenges, complicating access for both the general population and migrants [57]. Migrants must enroll in the statutory health insurance scheme for comprehensive access to medical services other than emergency care [58]. In South Africa, while primary mental healthcare is available to migrants, higher-level care is hindered by means of testing (which calculates the fee depending on the patient’s income), language barriers, and discrimination [59]. Furthermore, mental health services are primarily centralized in secondary hospitals and specialized facilities, with limited resources allocated to other levels of care, especially at the community level [60]. Macao (China) integrates mental health services within its broader healthcare system [61], but their effectiveness is limited by cultural factors and underdeveloped infrastructure [62]. Our findings align with previous reports from the European Union, which highlighted that only ten Member States have relevant national strategies or policies addressing migrants’ mental health, and just six include it under national health insurance [55]. These disparities underscore the need for targeted policies to improve mental healthcare access and inclusivity for migrants.

Finally, each of the five countries has become important destination countries for migrants in their respective regions. Germany currently has the largest foreign-born population in Europe [63]. At the same time, in the Netherlands, approximately one person in four was born abroad or has at least one parent born abroad [64]. Romania has become one of the fastest-growing destination countries for migrants in Central and Eastern Europe [46, 65], South Africa has become one of the most important host countries for migrants in Africa [47], and Macao (China) has become an important destination for international migrants from Southeast Asia [66].

## Methods

Informed by Bronfenbrenner’s Ecological Systems Theory [67], which posits that an individual’s development is influenced by their interactions within a series of nested environmental systems, this qualitative research delineates the multilayered interactions between migrants and the healthcare system, encompassing opinions of stakeholders from the microsystem, mesosystem, and macrosystem. Utilizing a purposive sampling strategy, participants from five distinct countries were engaged. Data were systematically gathered using semi-structured interviews and subsequently interpreted through thematic content analysis. The research employed a mixed deductive-inductive approach, anchored initially in a thorough review of existing literature on mental health care barriers for migrants. This foundation informed the development of semi-structured interview guides. This strategy allowed the research team to start with established evidence and then move beyond, uncovering new insights from the participants’ experiences and expertise. This research followed the COREQ (Consolidated Criteria for Reporting Qualitative Research) guidelines for qualitative studies [68].

Semi-structured interviews explored barriers that impact access and treatment of migrants with mental health needs in the healthcare systems of Germany, Macao (China), Romania, South Africa, and the Netherlands. Participants were selected through purposive sampling. The study involved 42 semi-structured interviews conducted between May and September 2021 with persons working in organizations and institutions that comprise the healthcare system and migrant support institutions in the selected countries (Table 1).

Participant selection was purposefully strategized to encapsulate three primary ecological levels. At the Macrosystem level, 14 individuals who exert influence on cultural values and policies were recruited. The Mesosystem was represented by 13 stakeholders functioning as intermediaries between individual experiences and larger societal structures. Finally, the Microsystem included 15 participants, comprising individuals with lived experiences and those who interact directly with them, including social and healthcare workers. Participants were identified and recruited through professional networks, ensuring a comprehensive representation of diverse perspectives across the ecological spectrum. (Table 1).

**Table 1:** Participants’ characteristics (*N* = 42)

No.	System Level	Occupation	Years in current role	Gender	Age
**Germany**					
1	Macro-level	Federal Migration and Refugee Official	26	Female	-
2	Macro-level	Physician, Medical Association Board Member	20	Male	51
3	Macro-level	Physician, Medical Association Board Member	30	Female	-
4	Meso-level	Chief Psychiatrist at Psychiatry Clinic	19	Male	49
5	Meso-level	Founder of Migration Integration Center, Social Worker	16	Female	50
6	Micro-level	Psychotherapist at Outpatient Mental Health Centre for Migrants	6	Female	59
7	Micro-level	Self-employed Interpreter	7	Female	56
8	Micro-level	Migrant with lived experience	-	Female	56
9	Micro-level	Migrant with lived experience	-	Male	30
**Macao (China)**					
1	Macro-level	Local Community Leader	22	Female	40
2	Macro-level	Legislative Council Member	5	Female	48
3	Meso-level	University Counselling Program Coordinator	25	Male	48
4	Meso-level	Board Member at Private Clinic	32	Male	69
5	Meso-level	NGO Executive Member	25	Female	48
6	Micro-level	Migrant Workers Union Representative	20	Female	40
7	Micro-level	NGO Staff Member	3	Female	25
8	Micro-level	Migrant Workers Union Representative	5	Male	45
**Romania**					
1	Macro-level	Health Ministry Official, Academic Lecturer	7	Male	35
2	Meso-level	Local Government Official, Academic Lecturer	3	Male	36
3	Meso-level	Hospital Psychiatry Unit Head, Academic Lecturer	12	Female	44
4	Micro-level	Social Services Psychotherapist at Refugee Agency	5	Female	27
5	Micro-level	Migrant with lived experience	-	Male	32
6	Micro-level	Former Social Worker at Human Rights NGO	2	Female	32
7	Micro-level	Social Worker at Human Rights NGO	2	Female	24
**South Africa**					
1	Macro-level	Government Health Researcher, Former Mental Health Department Director	30	Male	66
2	Macro-level	Founder & Chief Executive at Mental Health Peer Network	15	Female	48
3	Macro-level	Public Mental Health Researcher; Associate Professor, Deputy Director Public Mental Health Centre	21	Female	68
4	Macro-level	Head of Professional Psychiatry Organization; Associate Professor	12	Male	53
5	Macro-level	Public Health Specialist, Health Activist at University; Professor School of Public Health	25	Male	64
6	Meso-level	Mental Health Researcher, Psychologist, Specialist in Psychosocial Disability	14	Male	55
7	Meso-level	Senior Lecturer, Psychiatry and Mental Health Researcher, Expert in Language Accessibility	14	Female	56
8	Meso-level	Director at University Health Communication Center; Associate Professor Speech Therapy	11	Female	43
9	Micro-level	Clinical Psychologist, Health Researcher	3	Female	41
10	Micro-level	Coordinator at Migrant Support Organization, Refugee	8	Male	38
**The Netherlands**					
1	Macro-level	Human Rights NGO Campaign Leader	5	Female	53
2	Macro-level	European Commission Official	18	Male	45
3	Meso-level	Chair of Migrant Patient Organization	14	Male	60
4	Meso-level	Chair of Dutch/Moroccan Practitioners Organization; Medical Specialist	4	Male	35
5	Meso-level	Coordinator at Psychiatric Knowledge Center for Migrant Care	4	Female	43
6	Meso-level	Managing Director at National Psychotrauma Centre	6	Male	50
7	Micro-level	Transcultural Psychiatrist, Trainer of Working with Interpreters in Mental Healthcare	6	Male	46
8	Micro-level	Psychiatric Counselor	10	Female	22

All interviews were conducted by trained personnel proficient in both the native language of the participants and in English. Interviews were conducted via online platforms, telephone, or in-person in private settings, tailored to participant preferences and ensuring confidentiality and comfort. The duration of each interview varied between 30 min and 1 h. The interview questions focused on the general and language barriers for migrants who suffer from mental health problems concerning access and treatment in the healthcare system, the consequences of barriers, existing resources, and emergent practices employed to overcome the barriers they encountered, and their disadvantages and advantages (Table 2). Audio-visual data were collected during semi-structured interviews, transcribed verbatim, and personal identifiers removed for privacy. Transcripts were independently reviewed and translated into English by bilingual researchers to maintain the integrity and nuance of the original dialogues.

Data analysis was guided by the principles of thematic analysis, which was congruent with the framework proposed by Kuckartz [69]. During the planning phase of this research, we established a set of questions grounded in existing evidence related to mental health care access for migrants. This deductive phase laid the groundwork for our inquiry, guiding the adaptation of a thematic matrix specifically crafted to highlight the unique and common challenges encountered across different countries. To facilitate this process, we utilized the online platform Miro for collaborative mind-mapping sessions. These virtual workshops enabled our team to systematically identify, organize, and visually map the key themes and sub-themes relevant to each country, as well as those prevalent across multiple settings. Following this structured organization, we entered an inductive phase, aiming to delve deeper into the data to discover emergent sub-themes and coding patterns that were particularly informed by our participants’ insights. This phase allowed us to extend our analysis beyond the initial framework, uncovering nuanced aspects of the migrant healthcare experience. The organization and synthesis of our findings were meticulously conducted using Microsoft Office Excel, which served as an invaluable tool for managing our data efficiently and transparently. Within this structured environment, we carefully selected representative quotes that vividly illustrated each theme, thereby grounding our analysis in the authentic voices of our participants.

**Table 2:** Sample questions from Interview Guideline

What barriers are there for migrants who suffer from mental health problems concerning access and treatment in the health care system?
What barriers are specific for access and what are specific for treatment?
Are there any other barriers you can think of?
Now let us focus on language barriers for migrants who suffer from mental health problems. What role do you think language barriers play in the context of access and treatment in the health care system?
How prevalent are language barriers in the health care system?
What do you think are the consequences of language barriers concerning access to health care and treatment outcomes?

## Results

**Fig. 1:**
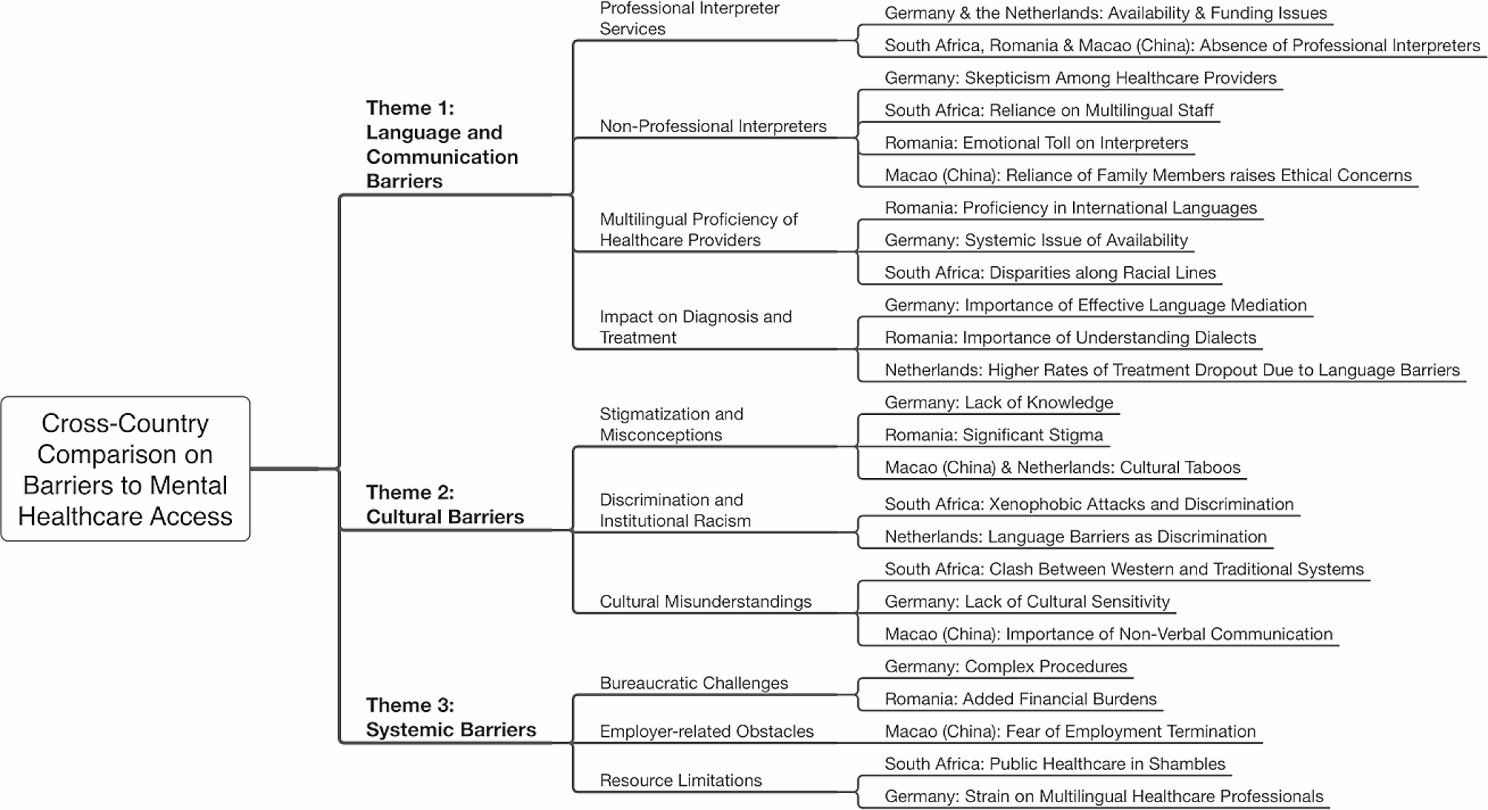
Identification of major and minor themes regarding barriers that emerged from the thematic analysis

The thematic analysis revealed three major themes: language and communication barriers, cultural and social barriers, and systemic barriers. The report highlights the main and subthemes that act as barriers in each country, which are presented in Fig. 1. This figure offers a summary of the most prominent themes that emerged in each country, along with specific details for each theme. However, it is important to note that this representation is not exhaustive and is intended to provide a general overview of the findings.

### Theme 1: Language and communication barriers

The analysis revealed that language discordance constitutes a major barrier in all countries. The lack of funding for professional interpreters and the lack of multilingual skills of healthcare professionals were common sub-themes. Various strategies are used to overcome language barriers, including professional and non-professional interpreters, digital tools, scarce language resources, and multilingual staff.

### Professional interpreter services: availability, use, and funding challenges

Across all countries, the inadequacy or complete absence of professional interpretation services in the healthcare system emerged as a common issue. This was especially pronounced in China, Romania, and South Africa, where no participant reported working with professional interpreters.

Germany and the Netherlands had professional interpreters available, but financial limitations hindered their widespread use. In Germany, one healthcare provider explained that the absence of legal regulations concerning interpreters in healthcare exacerbates the funding problem:

*“There is no regulation of interpreting. There is no government or cash health funding, which means it’s a funding problem [P04, Chief doctor (psychiatrist), Clinic for Psychiatry, Germany].”*

The financial barrier was also noted in the Netherlands as a significant issue. A participant stated:

*“The very basis, the crux, is that those interpreters are financed. Suppose there is no arrangement for using interpreters from a financial point of view. In that case, the financial aspect is already a barrier [P01, Human Rights NGO Campaign Leader, The Netherlands].”*

A participant in South Africa brought up the issue of insufficient funding for language services and how discriminatory attitudes affect the allocation of healthcare resources. This situation calls for a delicate balance to be struck in ensuring adequate interpreter services while not creating resentment among locals who may perceive migrants as being favored. It highlights the need to address both financial constraints and biases that hinder the fair distribution of services to all communities. This sentiment was captured in the statement:

“*What kind of resources should one be putting in / to ensure that (i.e., having suitably equipped interpreters? … people could get quite upset / if they felt that foreigners are given more services and more privilege than they are as South Africans [P01, Government Health Researcher, Former Mental Health Department Director, South Africa]*.”

In Romania, health institutions struggle to offer translation and interpretation services, especially for migrants who cannot communicate in any of the international languages spoken by the medical staff:

“*The problem comes from the inability of health institutions to offer translation services, for example, those migrants who do not speak any of the languages medical staff could handle [P01, Former State Secretary, Romania*].”

A social worker in China highlights this disparity through her personal experience:

“*Yes, but one time, the people in the hospital asked me [a social worker from an NGO], “Oh, can you help me to explain, to translate it to them.” And I said, “Isn’t it that you guys have an expert translator in the hospital? … And she answered me, “Huh?” (laughs). They don’t have, they [nurses and doctors in the hospital] have no idea… [P07, NGO Staff Member, Macao (China)]*.”

### Use of non-professional interpreters and associated challenges

The lack of professional interpreters has led to a reliance on non-professional interpreters or multilingual staff. In Germany, this expectation causes scepticism among healthcare providers, as noted by one interpreter:

“*There is no interpretation. They should always bring interpreters. […] Who always has the time to go along somehow every time? [P07, Interpreter, self-employed, Germany]*.”

In South Africa, healthcare providers often resort to using multilingual individuals available in the hospital, such as nurses, cleaning staff, security guards, or even family members. This practice, as one participant noted, raised concerns over professionalism and accuracy:

*“I think they / are not professionally trained to be translators or interpreters. They are used because they are accessible in the hospital and are able purely because they speak both languages [P05, Public Health Specialist, South Africa].”*

This approach raises concerns about the accuracy, professionalism, and confidentiality of translation. Another interviewee from the medical field pointed out that:

*“Employees are misused as language mediators or interpreters… it is not good [P01, Federal Migration, and Refugee Official Germany]”*, suggesting that using untrained staff as interpreters can jeopardize the quality of healthcare communication.

Similar situations are observed in the Netherlands and China, with an added concern about patient privacy, limitations in sharing necessary health information, and trust, as participants shared:

*“The patient’s personal story is hampered because the patient is afraid that his/her story will be known by all [P03, Chair of Migrant Patient Organization, The Netherlands].”*

“*If the interpreters or translators are from family members or their relatives, you may not be getting all of the information that you want; the family members maybe trying to hold back some of the information, some information that they don’t talk about in their culture … and if you’re dealing with someone from Asian culture and talking with family members or interpreters for an elderly patient, the family member may not want the elderly patient to know how serious the disease is [P05, NGO Executive Member, Macao (China)]*.”

Using children as interpreters was particularly problematic, leading to potential trauma, disruption of their education, and ethical issues, as one respondent from the Netherlands highlighted:

“*I’m baffled how very much - even caregivers who have worked a lot with migrants -, how very much they are just completely silent about what it does to a child when it has to interpret, for example, and that it impacts the quality, that it impacts the parent-child relationship, that the child doesn’t go to school [P01, Human Rights NGO Campaign Leader, The Netherlands]*.”

In Romania, a psychotherapist underlined the emotional toll on non-professional interpreters, stating:

*“Many times, I had to do a prior training with the translator, [followed by] another session… in which I was trying to ease him of that load [P04, Psychotherapist, Romania].”*

In Romania, one additional contribution to the lack of linguistic support is the missing assistance from embassies, as noted by one head of a psychiatric unit:

“*If the Embassy does not offer them support and does not offer us support to provide us with a professional translator to help us in the interview, then things get delayed, and the psychiatric consultation and the patient’s care are delayed [P02, Psychiatry Unit Head, Romania]*.”

The results show that using informal interpreters brings up concerns about the role of interpretation. As two respondents from South Africa explained:

“*People (interpreters) very often feel that ah, they are being extremely helpful to you, in saying more to the person, than you have actually said [P02, Founder and Chief executive at mental health peer network, South Africa]*.”

“*I think interpretation still doesn’t mean counseling [P05, Public Mental Health Specialist, South Africa]*.”

The interviews revealed an overarching lack of professional interpretation services across healthcare systems, leading to improvised, suboptimal, and potentially unethical solutions. The common issues identified were a lack of funding and regulations and the use of untrained individuals as interpreters. The use of non-professional interpreters is being deployed to address language barriers in healthcare settings across these countries, and drawbacks of this approach include confidentiality breaches, trust erosion, emotional distress, and ethical dilemmas, underlining the importance of adequately trained professional interpreters. The findings indicate a clear need for policies to regulate and fund professional interpretation services.

### Multilingual proficiency of healthcare providers

The multilingual proficiency of healthcare providers (HCPs) varied across countries, impacting patient care and presenting distinct challenges. These findings reveal disparities in healthcare communication that are influenced by language barriers, multilingual provider availability, and linguistic skills of healthcare providers. While certain strategies, such as consulting in English, have been proposed to overcome these obstacles, the effectiveness of these approaches remains controversial.

In Romania, mental health professionals typically possess proficiency in other languages, aiding communication with patients who can converse in these common tongues. As a psychiatrist stated:

*“I mean that mental health professionals are good speakers of languages of international circulation (…) We can handle people who come to the clinic and speak one of the languages of international circulation [P03, Psychiatry Unit Head, Romania].”*

Contrastingly, Germany faces a systemic issue regarding the availability of multilingual therapists. An interview participant voiced this challenge, saying:

“*In terms of access, it is still difficult that we have very few multilingual therapists [P01, Federal Migration and Refugee Official Germany]*.”

In South Africa, healthcare communication is uniquely impacted by the intersectionality of language, ethnicity, and race. A disparity in multilingual skills along racial lines is evident, with one participant highlighting that white professionals, who form a larger proportion of qualified practitioners, often lack fluency in local languages:

“*As we know, we still have far more / uhm / white people who are qualified and don’t speak many of the local languages [P02, Founder and Chief Executive at mental health peer network, South Africa]*.”

To mitigate language barriers, many South African HCPs propose using English as a lingua franca. However, this approach is contested by those who believe that patients struggle to express their health concerns fully in English. As one participant from South Africa stated:

*“My own language / I would be in a position to narrate and describe how everything started using English, it is very difficult for me to explain [P10, Co-ordinator, Migrant Support Centre and Refugee, South Africa].”*

This problem extends to the diagnostic process, where a participant emphasized the difficulty of accurate diagnosis with patients speaking broken English:

*“Although many patients can speak English, yet it is very much, you know, broken English (…) which interferes with gaining rich or very accurate information so that it becomes challenging to make a diagnosis [P08, Director at University Health Communication Centre; Associate Professor Speech Therapy, South Africa].”*

The Chinese context highlights substantial language barriers for both healthcare professionals and patients. A participant from China highlighted the difficulty of accessing mental health care due to language barriers:

“*So then if they want to access mental health care in Macao (China), they can’t really rely on the public medical services. It’s not available, and also the nurses, doctors, and they have the right not to speak English [P02, Legislative Council Member, Macao (China)]*.”

These barriers extend to non-native English speakers, who struggle to articulate complex health issues:

Moreover, the level of English proficiency varies among patients, with a participant from Macao (China) stating, “*…even if they speak well in English, usually maybe the middle school or primary school already they learn in the English sessions [P01, Local Community Leader, Macao (China)]*.”

### The impact of language barriers on diagnosis and treatment

This subtheme explores the detrimental effects of language barriers in healthcare, including misdiagnoses, treatment errors, and compromised quality of care. These barriers significantly challenge the healthcare providers’ ability to provide accurate diagnoses and effective treatments, particularly in Germany, Romania, and the Netherlands.

In Germany, the importance of effective language mediation in diagnosing health issues was underlined. As one professional stated:

*“Well, the moment I do not have a language mediation, I am limited in my possibilities. Yes, so I-, also the risk, I understand something wrong and therefore make a wrong diagnosis for example [P06, Psychotherapist, Outpatient Mental Health Centre for Migrants, Germany].”*

This quotation highlights the potential adverse outcomes of language barriers, where miscommunication could lead to incorrect diagnoses and improper care.

The same situation is emphasized by a Romanian participant, who highlighted the multifaceted impact of language barriers on the healthcare experience, particularly in psychiatric settings. A psychiatry unit head stated:

“*Linguistic barriers decrease patient and medical staff satisfaction and communication between medical providers and patients. Patients who face language barriers are more likely to consume more or fewer healthcare services and experience more adverse events. Patients experience poorer patient examination, misdiagnosis, delayed treatment, incomplete understanding of the patient’s condition, and low trust in services received [P03, Psychiatrist, Romania]*.”

These insights illustrate that language proficiency extends beyond speaking the same language—it also requires an understanding of regional language nuances.

In the Netherlands, language barriers are also seen as obstacles to the quality of treatment for migrant patients. HCPs expressed difficulties in establishing connections with patients and understanding their problems due to these barriers. As one participant shared:

*“In terms of language, I think it is also difficult. Because you, of course, already have a problem with making a connection with a client anyway. That is often difficult to make someone feel heard and really understand someone [P08, Psychiatric Counselor, the Netherlands].”*

This comment highlights the emotional strain language barriers can impose on HCPs who may feel unable to deliver optimal care due to these constraints.

Furthermore, patients themselves also grapple with expressing their concerns adequately, contributing to feelings of being unheard and misunderstood. Consequently, language barriers are associated with higher rates of treatment dropout or absences, as elaborated by one participant:

“*If you take a look at the group of people who actually have insufficient command of the Dutch or English language, but, because there is no other option, enter into treatment, you will see that the problem is you sometimes cannot properly complete treatment or go into deeper layers, because someone gets stuck in terms of the language. So, they cannot share their emotions well enough, cannot express sufficiently what is going on and then you often see a high dropout rate or high no-show [P05, Coordinator at Psychiatric Knowledge Centre for Migrant Care, the Netherlands]*.”

### Theme 2: Cultural barriers

Based on our analysis of interviews, we found that there are common challenges faced by healthcare professionals in providing mental health services to migrants. These challenges include language barriers, cultural differences, and a lack of cultural competence among healthcare professionals. These factors contribute to stigma, discrimination, and limited access to mental healthcare services for migrants. Our findings highlight the need to address these challenges by enhancing cultural competence among healthcare professionals and by raising awareness of mental health issues within migrant communities. We also found that cultural norms significantly impact healthcare expectations, which underscores the importance of providing culturally inclusive healthcare services to migrant populations.

### Stigmatization and misconceptions about mental health

The stigmatization and misconceptions about mental health are prevalent issues in many countries, affecting both the native population and migrant communities. During an interview in Germany, a participant pointed out that migrants frequently encounter obstacles in accessing mental healthcare due to the existence of different mental health models, stigmatization, and limited knowledge of their rights and the healthcare system. This implies that effective interventions should be culturally sensitive and strive to bridge these gaps in understanding while also educating migrants about their rights within the healthcare system:

*“A deficiency of knowledge about their rights and the healthcare system of the host country, stigmatization, and differing explanatory models of mental health disorders and expectations concerning treatment could also be a barrier [P05, Founder of Migration Integration Center, Social Worker, Germany].”*

It has been reported that both the general population and migrant communities in Romania face stigma when it comes to mental health. As the Former State Secretary noted:

*“Mental health care is often not regarded as a priority, and it is rarely spoken of” and “access to mental healthcare is also deterred by a certain degree of stigma associated with mental health affections in both the host country but as well within migrant communities and groups [P01, Former State Secretary, Romania].”*

Furthermore, in Romania, a former accredited social worker highlighted the stress and trauma experienced by migrants in navigating a new society and healthcare system. They stated:

“*Within the migrant and refugee groups that I worked with I could identify clear signs of trauma and mental health problems. But people were too worried about their material problems…that they would not have any bandwidth left to think about mental health problems [P06, Former Social Worker, Romania]*.”

Cultural taboos and the stigma related to mental health also emerged as significant barriers in China and the Netherlands. A participant from China stated:

“*Actually, there are many [mental problems], but they don’t want to bring it up … we should run to a psychologist, but yes, that’s the stigma that I don’t want people to see me in this way [P06, Migrant Workers Union Representative, Macao (China)]*.”

Similar sentiments were echoed in the Netherlands, where participants cited the cultural taboo surrounding mental health as a significant deterrent to some migrants seeking mental health care. This stigma was captured by P03’s statement:

“*When you start talking about mental healthcare, they [migrants] will be a bit distant and cautious…they will think ‘I am not crazy. Why should I go to treatment? [P03, Chair of Migrant Patient Organization, The Netherlands]*.”

Societal and cultural misunderstandings of mental health in Macao (China) result in systemic barriers. The societal dismissiveness of mental health needs, especially among the local population and employers, makes it difficult for migrant workers to seek help and be taken seriously:

*“The local population does not understand mental health enough. So then, they tend to ignore people’s need for this, and it’d be quite easy for them to, per se that, if the migrants say: “We need to, I need to, “they might just say that “Oh, you’re just homesick” or “you’re just pretending you’re sick” and any kind of situation like that. So, you can see that, on the one hand, we do not have enough service providers, then the language barrier, and then the social culture, you know, all people who kind of not know about mental health would also be a barrier for them [P02, Legislative Council Member, Macao (China)].”*

### Discrimination and institutional racism

In South Africa, interviewee P10 brought forward the issue of discrimination against foreign nationals, stating that being a foreigner can make it more challenging to receive treatment. P10 expressed:

*“Discrimination… that you’re foreign national/actually counts against you, to receive treatment… [P10, Manager, Migrant Support Centre, personal migrant background, South Africa].”*

In South Africa, there are documented xenophobic attacks on foreigners, and discrimination against foreign nationals affects their access to healthcare services:

*“You know, the minute they start interrogating people: Where do you come from? Uhm. Then, it is just xenophobic through and through. … So we know that xenophobia is really rife for all migrants who are accessing any kind of healthcare, social care … xenophobia is pervasive, and unfortunately, that is really ingrained in the healthcare system and also coming from healthcare practitioners themselves. // that xenophobia layer hangs around all the time, whether it is, uhm, in communities or in healthcare institutions [P08, Director at University Health Communication Centre; Associate Professor Speech Therapy, South Africa].”*

Discrimination and institutional racism also appeared in the Netherlands, where interviewees pointed out the difficulties non-native speakers face in accessing healthcare due to language barriers. P07, for instance, noted this situation as follows:

“… *a clear example of institutional racism [P07, Local Community Leader, The Netherlands].”*

### Cultural misunderstandings and inadequate services

In South Africa, cultural misunderstandings between the Western bio-medical model and traditional health systems were significant. Interviewee P03 highlighted the lack of a common understanding of what constitutes mental health and mental illness:

*“So, the cultural understanding of mental illness and what counts as that (differs among different communities of users) [P03, Public Mental Health Researcher; Associate Professor, Deputy Director Public Mental Health Centre, South Africa].”*

Another participant revealed the limited consideration given to refugees’ cultural beliefs and traditional practices in public health services, stating:

*“Public health services are rarely tailored to consider // cultural beliefs and traditional practices of refugees or asylum seekers. At home, HCPs may encourage patients with mental health issues to see a traditional healer. [P10, Manager, Migrant Support Centre, personal migrant background, South Africa].”*

Healthcare providers’ unpreparedness to cater to migrants’ unique needs is another recurring theme in the study. In Germany, one interviewee lamented that healthcare providers seemed ill-equipped for the distinct treatment requirements of these populations:

*“The doctors in private practice are not prepared for the structures that the refugees bring with them [P02/P03, Physician, Medical Association Board Member, Physician, Medical Association Board Member, Germany].”*

For an effective treatment process, HCPs should be culturally sensitive and open to understanding different cultures, mental health customs, and languages. P05 from the Netherlands acknowledged that culturally sensitive providers deliver better results:

“*What you see is that when providers are culturally sensitive, you see that the results are already much better. Even if the language is not optimal and patients do speak sufficiently Dutch or English, treatment can sometimes be completed [P05, Coordinator at Psychiatric Knowledge Center for Migrant Care, The Netherlands]*.”

In China, non-verbal communication is often used to express emotion and convey meaning. This can be a barrier to effective communication with healthcare providers who may not be familiar with these cultural norms. In the Asian context, migrants may not express things directly, and their concerns manifest through non-verbal communication:

“… *That’s the one that comes to mind most often, and /um/, others would be that um, the non-verbal communication from the different cultures maybe, maybe different and so if someone in particularly in Macao working from an Asian culture, they generally are not taught or not as adept at picking up non-verbal cues um from the patients such as, the um anxious movement that the patient may have, the lack of eye contact the patient may give (…) [P05, NGO Executive Member, Macao (China)]*.”

### Theme 3: systemic barriers

Whilst the language and cultural barriers are built-in and dealt with at an interpersonal level, the research also taps into the systemic barriers that stem from how healthcare systems are designed in each national context we are analyzing.

### Bureaucratic challenges and cost implications

In Germany, the healthcare system presents its own complex and time-consuming bureaucratic procedures. As one interviewee notes, the healthcare system’s complex and time-consuming procedures can impact anyone seeking care from the general population, including migrant communities:

“*Even if you or I am now in therapy or have psychological problems. You have to be very active about it. You have to go yourself and say to the family doctor: I have problems here. The general practitioner has to recognize it and be prepared to refer you. You have to sort things out with the health insurance company. You have to see if you can find a therapist who suits you [P01, Federal Migration and Refugee Official, Germany]*.”

Romania faces similar issues, but with added financial burdens. Migrants must navigate a complex and costly process to gain health insurance and access to healthcare services. The complexity of this process, combined with the language barrier and limited resources, makes it particularly challenging for many migrants:

*“When it comes to registering for health insurance and access to healthcare, entering the healthcare system is complex, costly, and bureaucratic” and “this process can be not only complicated but also costly for many migrants who recently entered Romania, do not speak the language, and have scarce financial resources [P06, Former accredited social worker, Romania].”*

As summarized by one Romanian interviewee, the specific problems are:

“*The ability of migrants to be covered with insurance, to have access to health services, to have sufficient knowledge about how the health system works, where to address when they have health problems, language barriers, trust in the health system and the ability to know to whom to address and from whom to take a prescription [P01, State Secretary, Romania]*.”

The issue of cost is further exemplified in the Romanian context, where the fee for enrolling in the healthcare system may be equivalent to the handout allowance provided for monthly expenses. This, along with additional monthly fees after the first six months, is a significant barrier to healthcare access for refugees and migrants:

*“The cost for enrolling in the system now is around 1300 Romanian Lei (around 275 euros at the time of the interview). Once we enrol them and we pay this amount they are enrolled for 6 months, and they have full coverage. This often equals the handout allowance that they are offered for groceries and monthly expenses [P06, accredited social worker working in integration services, Romania].”*

### Employer-related obstacles

In Macao, China, fear of employment termination acts as a barrier to migrants seeking healthcare services. Further, the lack of enforcement of the requirement for employers to pay for a minimum insurance policy for migrant workers leads to limited access to healthcare services:

“*The problem is that the patient [migrant worker] is not allowed by the employer to return for multiple visits even if we [NGO clinic] don’t charge for that (…) the big problem is that the employer doesn’t really follow the law and pay the insurance policy for the patients [P05, NGO Executive Member, Macao (China)]*.”

Language barriers, specifically in Macao, China, also exacerbate systemic challenges to mental healthcare access. Documents provided in Portuguese, a language many migrants are not familiar with, create additional obstacles for them:

“… *and the paperwork right here is in Portuguese, and it is difficult, because I have zero ability in Portuguese, for example, like … it still can be, but the writings here are in Chinese - Portuguese, and that is the obstacle [P08, Migrant Workers Union Representative, China].*”

*“Sometimes if we go to the doctor, the receipt is given, and maybe there is a pharmacist, but we consume the medicine based on its dose in number [P08, Migrant Workers Union Representative, Macao (China)].”*

### The lack of resources for mental healthcare services

In Germany, there is a critical strain on multilingual healthcare professionals who are often migrants themselves. Due to the influx of requests, these practitioners, including doctors in private practice and those in specialized facilities like treatment centres for torture victims, are often overwhelmed:

*“But these are often practitioners who themselves have a migration background and who develop a great passion for this topic. But they are also often overwhelmed because they have to deal with so many requests… It’s a huge burden for the treatment providers to have to turn people away…And I think that is what also wears the practitioners down a bit [P01, Federal Migration and Refugee Official, Germany].”*

In South Africa, access to mental health services is a major concern as limited services are available, making it difficult for those in need to receive the required support:

“*But then, uhm, there’s the same barriers as people in South Africa have in accessing services because there aren’t enough services / for the local population / anyway [P01, Government Health Researcher, Former Mental Health Department Director, South Africa].*”

This was also echoed by P08:

“*It’s not just access for migrants … one could say that it is pretty much in shambles … unfortunately …. (for all) accessing public healthcare, mental healthcare systems… [P08, Director at University Health Communication Centre; Associate Professor Speech Therapy, South Africa].*”

In China, there are systemic barriers to reaching migrants, as this is done through passive means such as posters and fliers. The approach towards change is defensive and resistant, with no acknowledgment of clinical psychology and mental health treatments.

“*So, I think that in Macao, everyone says the barriers which we don’t have enough mental health services which are we do not have enough, so then this is kind of barrier for everyone, but for the migrant workers, there would be an extra barrier so aside from they have to access the mental health service and the language is a bit issue. On the one hand, all kinds of local information are not always- it’s not easy to find, you know, social services information in English. So, most of the things here, this is a Chinese city, and even though we believe this is also an international city, the official languages are Chinese and Portuguese [P06, Migrant Workers Union Representative, China]*.”

The healthcare system in all five countries aims to reach universal coverage. However, it fails to grasp the extent to which migrants need better access to mental healthcare services and plan resources and strategies to meet this need. In countries like China, the Macao Special Administrative Region, Romania, and South Africa, the urgency of addressing migrants’ mental healthcare needs is even less acknowledged.

## Discussion

The objective of this study was to elucidate the barriers to healthcare access encountered by migrants in Germany, Macao (China), Romania, South Africa, and the Netherlands, with a particular focus on mental health services. The data presented reinforces the complexities tied to healthcare access among migrants, substantiating earlier work that emphasizes the multifaceted barriers to healthcare access [29, 70, 71]. This study adds to the existing literature by comparing and contrasting healthcare barriers across countries with varying healthcare infrastructures and examining these issues through the specific lens of mental health services.

In our examination of the interplay between migrants and healthcare systems across diverse contexts, we uncover the nuanced bidirectional dynamics that underpin access to mental health care, as conceptualized within Bronfenbrenner’s ecological framework. Our findings highlight the microsystem interactions, particularly the pivotal role of language services, where the absence or inadequacy of professional interpretation directly impedes migrants’ healthcare experiences. Simultaneously, migrants’ encounters and coping mechanisms in response to these barriers illuminate the critical need for systemic change, potentially influencing policy revisions and resource allocation towards more accessible interpreter services. This interconnection extends to the mesosystem, where the reliance on non-professional interpreters not only compromises care quality but also catalyzes dialogue within healthcare institutions about enhancing language support, evidencing the reciprocal influence between individual experiences and organizational practices. At the broader macrosystem level, cultural perceptions and policies significantly shape the mental healthcare landscape for migrants, with stigma and policy deficiencies serving as formidable obstacles. Yet, migrants’ active participation in mental health discourse and advocacy heralds a gradual cultural shift towards inclusivity and recognition of their healthcare rights. Our research delineates the complexity of these ecological interactions, advocating for a multifaceted approach to healthcare policy and practice that acknowledges and leverages the dynamic exchanges between migrants and their environmental contexts. By foregrounding these bidirectional influences, we aim to contribute to the development of more responsive, equitable, and culturally competent mental healthcare systems that not only address the immediate needs of migrants but also adapt to the ongoing changes within global migration patterns and societal norms.

### Overcoming language and communication barriers

A salient finding was the role of language barriers in affecting the quality of healthcare service delivery. Previous research has established that language barriers negatively impact patient satisfaction, healthcare delivery, and the cost and quality of healthcare services [26, 71–73]. The finding that language barriers are a key challenge for migrants accessing healthcare services is consistent with earlier work [26, 29, 71, 72]. This study corroborates these findings and extends our understanding by exploring the effects in the context of mental health services for migrants in five different countries, thus adding a transnational lens to the issue. Lack of funding for professional interpreters emerged as a common issue, exacerbated by healthcare professionals’ limited multilingual skills. Language barriers were overcome using professional and non-professional interpreters, digital tools, language-reduced resources, and multilingual staff. As revealed by this study, Romania, China, and South Africa face particularly significant inadequacy and a lack of professional interpretation services within their healthcare systems.

The research indicates that professional interpreters are underutilized or unavailable despite the recognized necessity. Professional interpreters are available in Germany and the Netherlands, but financial constraints prevent their widespread use. This issue is further exacerbated by the lack of legal regulations governing interpreters in healthcare in Germany [74]. The Netherlands also faces financial challenges in funding interpreters. In South Africa, insufficient funding for interpreting services makes it difficult to provide care to linguistically diverse migrant populations, leading to concerns about unequal access to healthcare. Health institutions in Romania and Macao also struggle to provide interpreting services, posing significant challenges in ensuring effective communication and access to mental healthcare for migrants. Hence, addressing language barriers is critical for migrant patients to improve healthcare access and delivery, particularly to mental health services. A systematic review from 2018 [39] identified the key strategies to address communication barriers in healthcare settings for migrants —cultural mediation, interpretation, health information translation, multilingual healthcare providers, and healthcare provider training—that have shown a positive impact in improving health knowledge and access. However, gaps in training and role clarity for cultural mediators and interpreters indicate the need for standardization [39, 75, 76]. Klemm et al. (2015) distinguished between two important aspects of effective communication in healthcare involving migrants: linguistic interpretation and cultural mediation. Linguistic interpretation is the process of accurately conveying the meaning of language during interpersonal interactions. It is a professional activity and involves adhering to recognized standards and codes of conduct [77]. On the other hand, cultural mediation aims to bridge the gap between the ways of thinking and non-verbal communication of health workers and migrants. It helps both parties gain a deeper understanding of each other’s cultures and meanings, thereby fostering mutual understanding. Cultural mediators inform health professionals about the cultural practices of migrants and help migrants navigate the health system while also informing them about their entitlements [78].

The findings of the current study highlight the significant reliance on non-professional interpreters or multilingual staff in the healthcare systems of the studied countries due to the lack of professional interpreters. Despite the recognized need for interpretation and cultural mediation services, professional interpreters have often not been utilized adequately, largely due to migrant patients’ preferences, financial constraints, and systemic issues [29, 49, 50, 76, 79, 80]. The use of untrained individuals as interpreters poses significant risks to the quality of healthcare, introducing concerns about accuracy, professionalism, and confidentiality, an issue also raised by earlier studies [81–83]. The Global Competency Standards for Health Workers in Refugee and Migrant Health, developed by WHO’s Health and Migration Program in 2021, offer a framework for handling the language and communication barriers that exist between health workers and migrants. The document emphasizes the need for safe and effective language and communication aids, including interpreters and cultural mediators, to meet the unique requirements of these populations. These aids are crucial for bridging language and cultural gaps, improving patient comprehension, and enhancing the quality and safety of healthcare. The document also discusses the difficulties and ethical concerns associated with non-professional interpretation and points out that machine-automated translation technologies can be used as supplementary aids, even though they have certain limitations [41]. Efforts to tackle language barriers in healthcare should be evidence-based and involve diverse stakeholders, including patients and their families. These initiatives should be tailored to local contexts and consider ethical factors. Adopting a community-based research approach can facilitate co-created solutions that meet various needs, reduce stigma, and empower all participants in linguistically diverse healthcare interactions [84].

Additionally, the use of electronic translation tools has been proposed as a potential solution to overcome language barriers in healthcare settings. These tools may provide various benefits, including shorter consultation times, decreased reliance on interpreter services, lower patient anxiety, and favorable outcomes in terms of both acceptance and effectiveness [85]. Nevertheless, they also have notable disadvantages, such as inaccuracies in translation, possible misinterpretations arising from context-specific linguistic subtleties, and constraints in establishing the therapeutic relationship [86]. Ethical concerns about the inclusion of electronic translations in clinical interactions include issues of confidentiality, the accuracy of translated information, and the potential for compromised interactions [41]. Electronic translation tools can be helpful, but machine-automated translation is not very effective when dealing with complex and nuanced information, especially in health and legal contexts [41]. A lack of binding standards for assessing quality criteria and indicators when testing technological tools to overcome language barriers in healthcare has been reported [86].

Language barriers emerged as a predominant challenge in our study of healthcare access for migrants in China, Romania, South Africa, Germany, and the Netherlands, significantly impacting mental healthcare. Historical migration flows and linguistic landscapes in these countries have shaped their current healthcare challenges. In China, regions like Macao exhibit extreme multilingualism, with numerous Chinese dialects and foreign languages such as Portuguese, English, and Filipino, requiring comprehensive language support services [87, 88]. In South Africa, nationals from over 100 countries now reside there, with migrants from Nigeria, Ghana, Senegal, Mali, the Democratic Republic of Congo, and Zimbabwe, necessitating healthcare systems that can accommodate diverse linguistic needs [89]. Despite the country’s 11 official languages, English remains the dominant language in business, public life, and increasingly in domestic settings [90]. However, many migrants and citizens may not be proficient in English or any of the other official languages, posing significant barriers to healthcare access. Germany and the Netherlands face significant challenges due to large migrant populations from Syria and Turkey. Germany also sees substantial migration from Romania and Poland, with recent increases in asylum seekers from Afghanistan and Turkey [91]. The Netherlands similarly deals with an influx from Syria, Poland, and Romania, requiring targeted language services within their healthcare systems [91]. In Romania, the primary issue is the linguistic integration of a largely Romanian-speaking population with minority languages such as Hungarian and German [92]. The recent influx of Ukrainian refugees has amplified linguistic challenges, emphasizing the need for inclusive language policies to ensure effective communication and care [91]. While language barriers are often more immediate and major obstacles, emphasizing the importance of language support to ensure equal access to healthcare for migrants, other challenges, such as cultural aspects or power imbalances, should also be considered.

### Mental health issues, cultural barriers, and healthcare provider competence

The current research highlights issues with stigma, discrimination, and a lack of cultural competencies among healthcare professionals, and the fact that mental health care for migrants is a complex issue due to cultural barriers. These factors have been found to limit access to mental health services, creating significant barriers for migrants seeking healthcare.

Addressing mental health in migrants is burdened by both cultural and systemic factors [14]. The prevalence of mental health conditions is higher in these groups compared to the general population, yet diagnoses by healthcare providers are often missed [93]. People who migrate often face challenges associated with leaving areas of war, mistreatment, and financial difficulties. When they reach their destination, they might confront difficulties due to differences in language, lack of social support, and the necessity to adjust to new cultural and religious customs. These circumstances can negatively impact their emotional well-being [14]. Mental health stigma is a common issue across cultures, with some viewing mental health/illness as something to be ashamed of, taboo, or linked to evil spirits. This kind of stigma can act as a barrier for migrants to access mental healthcare. Moreover, the stigma can emerge from society, family, healthcare professionals, or within the individual themselves [94, 95]. Migrants encounter systemic obstacles when accessing mental health services due to lack of knowledge, communication difficulties, and fear of deportation. Disparities in care for minorities, migrants, and refugees highlight the importance of improving accessibility and adapting systems, services, and interventions [14, 96].

To support migrant and refugee patients, healthcare providers need training in cultural diversity and cultural competence. This includes addressing racial discrimination and providing resources like mental health professional skill-building [96]. Healthcare providers should receive guidance and training to develop the necessary skills, which includes training in cultural competence, working with interpreters, and understanding cross-cultural mental health. Additional support is offered through cultural consultancy services that help clinicians navigate intercultural challenges and improve their knowledge of cultural anthropology [39].

One important consideration in the provision of mental health care to migrant populations is the potential limitation of conventional mental health interventions, which often emphasize verbal therapies [97]. While these approaches can be effective for many, they may not adequately address the needs of individuals who face language barriers. The applicability of these psychotherapy models in diverse cultural contexts is a subject of discussion, underscoring the necessity for further research and the development of localized psychotherapy models [98]. Migrants, in particular, may require more innovative and culturally sensitive approaches to mental health care [99–103].

### Systemic barriers and policy considerations

The results obtained from the study indicate that there are various systemic barriers to mental health care for migrants, which are complex and intertwined. Barriers such as bureaucracy, finances, navigating the healthcare system, and lack of providers prevent migrant patients from accessing mental healthcare. The above-mentioned barriers highlight the urgent need for systemic and policy changes in these countries to ensure that mental healthcare access is inclusive, accommodating, and effective for all populations, including migrants. The changes must address bureaucratic hurdles, financial constraints, and a lack of resources for mental healthcare services to eliminate the complex interplay of economic, language, and cultural barriers that migrants face in healthcare settings. A scoping review from 2018 outlined several strategies to improve healthcare communication with refugees and migrants. However, the review also highlighted the need for more research to establish their effectiveness, indicating a lack of evidence-based knowledge regarding resources needed to establish and maintain the implementation of identified strategies [39].

Healthcare systems might be hard to navigate and seem overly bureaucratic, even for nationals who speak the official dominant languages of the system. In relation to migrants in vulnerable situations who are less proficient in the official language(s) of the system, the healthcare system is often outwardly repelling due to legal and bureaucratic barriers [10, 104]. Migrants frequently encounter various systemic barriers that limit their access to essential health services. These barriers may include institutional discrimination, inadequate health literacy, and limited access to mainstream services. To ensure equitable access to healthcare services for this population, it is necessary to implement a multi-faceted approach that involves policy changes, community engagement, and capacity building among healthcare professionals. While healthcare professionals play a critical role, it is also important to consider policy and legal frameworks that govern access to health services for migrants and refugees. National and international laws should be aligned with healthcare goals to ensure these services are effectively delivered [105].

One of the key strategies is to engage vulnerable migrants in their healthcare through community involvement. This approach was used by the MyHealth European project aimed to improve healthcare access and quality for migrants in Europe. The project proposed that local authorities should invest in health professionals’ cultural competencies and that migrants should adapt their help-seeking behavior [106]. To address healthcare inequalities, it is necessary to implement systemic changes through legislation. Healthcare institutions should also introduce intercultural mediators, provide cultural competence training for healthcare workers, and use a self-evaluation tool to measure the level of equity. Slovenia’s “Together for Health - Skupaj za zdravje” project, led by the National Institute of Public Health, utilized this approach effectively [107].

To improve healthcare access for migrants, healthcare policies and legal frameworks need to address systemic barriers that significantly affect their health. In many cases, these policies can lead to limited healthcare access and have been shown to affect mental health and contribute to health inequalities negatively. This suggests the need for more inclusive and migrant-friendly policies to improve health outcomes [108]. Migrants often face limited healthcare access due to their citizenship status. Many countries link healthcare access to citizenship, leaving migrants vulnerable when their claims are denied. Policies should prioritize the right to healthcare, regardless of citizenship status [109]. Migrants need to be made aware of their legal entitlements to improve their access to healthcare. Policies should include provisions for raising awareness among them [11].

### Limitations of the study

It is important to note that Bronfenbrenner’s framework has some limitations. The framework may overlook certain phenomena that do not fit the structured layers. There are inherent risks of selection bias and limited generalizability due to purposive sampling techniques, especially when recruiting participants through professional networks. Additionally, semi-structured interviews may introduce interviewer and respondent biases, which can be exacerbated by using different interview mediums. Transcription and translation may also lead to the loss of linguistic and cultural nuances even when proficient bilingual researchers are involved. Thematic analysis is subjective, and emergent themes that are not identified in the pre-constructed matrix may be excluded. There are also temporal constraints, as the study was conducted in 2021, which may affect the long-term applicability of the findings. Finally, ethical challenges may arise when discussing sensitive mental health topics, despite efforts to ensure participant confidentiality. The generalizability of our findings, rooted in qualitative methodology, does not extend to the healthcare systems of the countries studied in their entirety. This approach, pivotal for hypothesis generation, offers a nuanced understanding of targeted phenomena within healthcare contexts. It is important for readers to bear these constraints in mind when interpreting the findings and to recognize the potential for broader explorations in future research.

### Implications and future directions

Our investigation delineates the complex healthcare barriers migrants encounter, reinforcing the WHO’s advocacy for robust, systematic data collection to inform nuanced, evidence-based healthcare policies [108]. The study’s cross-cultural comparison suggests that overcoming these challenges necessitates a collaborative, intersectoral strategy, fostering healthcare systems that are both inclusive and responsive to the cultural and linguistic diversity of migrant populations. For example, while language barriers are a universal issue, the extent to which countries provide and fund professional interpretation services varies significantly. Germany and the Netherlands, with more established systems for interpretation, contrast sharply with South Africa and Romania, where financial and systemic constraints impede such services. This disparity underscores the potential for policy learning and adaptation across countries, where best practices in one context could inform policy developments in another. This approach emphasizes the potential of international collaboration and policy innovation, advocating for the adaptation of successful strategies across borders to enhance global migrant healthcare access. The diversity observed in healthcare experiences and systems across the studied countries highlights an opportunity for mutual learning and strategy adaptation, emphasizing the importance of creating flexible, culturally sensitive healthcare solutions to meet the diverse needs of migrants effectively.

The existing situation, as portrayed by this study, necessitates immediate and future interventions. Policymakers should consider providing sustainable funding options for professional interpreting services and for capacity building for healthcare providers. Considering these findings, it is imperative that policymakers, healthcare organizations, and mental health professionals in these countries take immediate and concerted action to address these barriers. This includes investing in professional interpretation services, enhancing cultural competencies among healthcare providers, combating discrimination and institutional racism, simplifying bureaucratic processes, and increasing resources for mental healthcare services. Only through collaborative efforts can these countries ensure that all individuals, regardless of their migration status or background, have equitable access to quality mental health care. Failure to address these issues not only perpetuates the suffering of migrants but also poses public health risks by leaving mental health conditions untreated and unmanaged. Simply relying on emergent solidarities that compensate the failures of the system at the fringes of the healthcare system does not represent a viable policy for the future.

## Conclusion

The present study highlights the various obstacles that migrants face when trying to access healthcare, particularly in mental health services. Our findings suggest that a combination of measures is needed to overcome these obstacles, including improvements in language services, capacity building among healthcare providers, and policy reforms. As global migration continues, it is essential that these strategies are urgently implemented as a fundamental part of healthcare service provision and are brought into the political agenda. Our research emphasizes the need for collaborative efforts among policymakers, healthcare organizations, and professionals to address these challenges. Neglecting to act in a timely manner could lead to significant public health risks and perpetuate the suffering of this vulnerable population.

## Data Availability

The datasets used and analyzed during the current study are available from the University of Amsterdam, Babeș-Bolyai University, Stellenbosch University and the University Medical Center Hamburg-Eppendorf. Data are available from the authors upon reasonable request and with the permission of The University Medical Center Hamburg-Eppendorf. Please contact the corresponding author and research project coordinator, Mike Mösko, for data requests and inquiries.
